# Mixed Micro/Macro Cache for Device-to-Device Caching Systems in Multi-Operator Environments

**DOI:** 10.3390/s24144518

**Published:** 2024-07-12

**Authors:** Minjoong Rim

**Affiliations:** Department of Information and Communication Engineering, Dongguk University, Seoul 04620, Republic of Korea; minjoong@dongguk.edu; Tel.: +82-2-2260-3595

**Keywords:** D2D caching, wireless caching, micro caching, multi-operator, hit ratio, offloading

## Abstract

In a device-to-device (D2D) caching system that utilizes a device’s available storage space as a content cache, a device called a helper can provide content requested by neighboring devices, thereby reducing the burden on the wireless network. To enhance the efficiency of a limited-size cache, one can consider not only macro caching, which is content-based caching based on content popularity, but also micro caching, which is chunk-based sequential prefetching and stores content chunks slightly behind the one that a nearby device is currently viewing. If the content in a cache can be updated intermittently even during peak hours, the helper can improve the hit ratio by performing micro caching, which stores chunks that are expected to be requested by nearby devices in the near future. In this paper, we discuss the performance and effectiveness of micro D2D caching when there are multiple operators, the helpers can communicate with the devices of other operators, and the operators are under a low load independently of each other. We also discuss the ratio of micro caching in the cache area when the cache space is divided into macro and micro cache areas. Good performance can be achieved by using micro D2D caching in conjunction with macro D2D caching when macro caching alone does not provide sufficient performance, when users are likely to continue viewing the content they are currently viewing, when the content update cycle for the cache is short and a sufficient number of chunks can be updated for micro caching, and when there are multiple operators in the region.

## 1. Introduction

Wireless data requirements are increasing rapidly due to the rise of high-definition video streaming services over wireless networks [1,2,3]. To meet the desired data requirements of all users, it is necessary to increase the wireless capacity per unit area, and efforts are underway to install more cells and increase the wireless capacity of each cell [4,5]. In recent years, massive multiple-input multiple-output (MIMO) technology has increased spectrum efficiency and the development of technologies using high-frequency carriers has led to a rapid increase in the available wireless bandwidth [6,7,8]. For example, in terms of maximum data rates, 4G is targeted at 1 Gbps, 5G at 20 Gbps, and 6G aims at up to 1000 Gbps [9,10,11,12]. However, there are significant form factor and cost challenges in applying massive MIMO technology to base stations using low-frequency carriers, and base stations using high-frequency carriers have coverage issues, leaving many areas in the shadow of high-frequency carriers unless a sufficient number of base stations are installed [13,14,15]. In addition, the maximum wireless data rate may exceed the capacity of the existing wired backhaul, so unless the base stations are equipped with ultra-high speed wired backhaul, large capacity may not be available due to the backhaul bottleneck [1]. As a result, the wireless capacity available in different regions can vary widely. Also, as cells get smaller, the averaging effect disappears, and the number of devices and data requirements in each cell can vary significantly. 

High-definition video streaming services account for a large and growing share of wireless data capacity. One of the other directions to address the exploding wireless data requirements is the use of video content caching systems [16,17,18,19]. A device-to-device (D2D) caching system, which uses a device’s available storage space as a cache, reduces the load on wireless networks by allowing devices to store content in their caches and deliver content to other devices using D2D communication when the content requested by a nearby device is in the cache [20,21,22,23]. 

D2D communication can use WiFi Direct or 5G New Ratio (NR) sidelink. WiFi Direct uses an unlicensed spectrum, and NR sidelink can use an in-band licensed spectrum, out-band dedicated spectrum, or unlicensed spectrum [24,25,26,27]. When D2D communication does not use an in-band licensed spectrum, there is no interference with cellular communication. 

In this paper, a device that stores content and delivers it to other devices using D2D communication is called a helper, and a device that receives content from a helper is called a user equipment (UE) [28]. A device can be both a UE that receives content from another device and a helper that provides content to other devices.

For a device to act as a helper, it must have sufficient storage space and be less power hungry to support continuous D2D communication. Not all devices can act as helpers because typical devices may not have enough free storage space and may have power consumption, security, or copyright issues [28]. Therefore, the number of UEs may be larger than the number of helpers, and multiple UEs may be associated with a single helper, forming a star topology. If the D2D communication does not use the in-band spectrum, helpers can also provide content to UEs of other operators, thus increasing the utility of the caches when there are multiple operators [29,30]. 

A content cache is similar in concept to a computer’s cache. A computer’s cache is based on temporal and spatial locality, with the assumption that data used once is likely to be used again. Similarly, if it can be assumed that popular content can be used repeatedly by multiple users, offloading can be achieved by storing popular content in the cache [31,32,33]. Not only should content be stored before peak hours, but even during peak hours; if there are intermittent periods when data demand is not high and additional data supply is possible, the cache can be updated to reflect real-time changes in popularity. In this paper, content-based caching based on content popularity is referred to as macro caching. However, macro caching alone may not be sufficient to achieve the desired offload performance, because users have different tastes and preferences, and the amount of actual video content is almost infinite, while the amount of free storage on a device may be small.

Various techniques are used to improve macro caching performance. If the mobility of users can be predicted by analyzing their movement patterns or schedule management, it is possible to predict which users will go to a congested cell and store content for them in advance [34,35,36,37]. If we know the social relationships between users, we can predict which UEs will be near a helper, and macro caching performance can be improved by storing content preferred by these UEs [38,39,40]. If users with similar interests are grouped together and encouraged to engage in D2D communication, the effectiveness of the cache can be increased because content stored for oneself can be used for other users with similar tastes [41,42]. 

When a device has a recommendation system, many users tend to choose from the recommended content [43,44]. The effectiveness of the cache can be increased if recommendations are made from cached content by jointly optimizing a caching system and a recommendation system [45,46,47], or if caching is performed taking into account the recommendation system [48]. 

Although these methods can increase the effectiveness of macro caching, it may be difficult to overcome the fundamental problem of macro D2D caching, which is that a device’s cache can only store a very small fraction of the total content. In this paper, we consider a caching method called micro caching, which has a different approach than macro caching. Micro caching is chunk-based sequential prefetching and stores content chunks slightly behind the one that a nearby device is currently viewing. We discuss how to improve the offload performance by allowing a helper to update its cache based on the content chunk viewed by nearby UEs instead of updating its cache based on content popularity, assuming that the helper’s operator is intermittently under low load even during peak hours. When D2D communication does not use an in-band spectrum, a helper can provide content to UEs from different operators, which can improve the performance of micro caching. We discuss the effectiveness of micro caching when there are multiple operators, devices from different operators are capable of D2D communication, and the operators are independently under low load. 

The micro D2D caching method proposed in this paper does not conflict with or compare with existing macro D2D caching methods, but can be used in conjunction with them, and the performance can be further improved using various existing techniques. The contributions of this paper are as follows:

(1) While most of the literature related to content caching uses content-based methods, this paper considers micro caching, which is chunk-based sequential prefetching.
(2) While many studies improve performance by considering social relationships, mobility patterns, recommendations, etc. based on content popularity, this paper considers micro caching which does not consider content popularity.
(3) This paper discusses how performance improvements can be achieved when the cache content can be updated intermittently during peak hours.
(4) Micro caching is not always better than macro caching, so this paper considers mixed caching, where a cache space is divided into micro and macro cache areas. This paper also discusses under what conditions and in what proportions the two cache areas are divided.
(5) This paper discusses how micro D2D caching can be utilized when there are multiple operators in a region and a helper can also serve content to UEs belonging to other operators.

This paper is organized as follows. Section 2 introduces micro D2D caching and describes how micro D2D caching works in single-operator and multi-operator environments. In Section 3, we discuss the proportion of the micro D2D cache area when a cache area is divided into micro and macro cache areas. Section 4 discusses the usefulness of micro D2D caching through numerical results and the percentage of the micro D2D cache area in various situations. Finally, conclusions are drawn in Section 5. 

## 2. Micro D2D Caching

### 2.1. Micro D2D Caching

Computer caches can use the concept of sequential prefetching to increase efficiency, as well as the notion that data used once can be used again. In applications where data is used sequentially, such as filters, data that is expected to be used in the future can be retrieved in advance, stored in the cache, and used when needed. The same concept can be used to cache video content. 

When streaming videos on YouTube, Netflix, etc., users do not retrieve the video content all at once, but in small chunks or segments of a few seconds as they watch the video. The Hyper Text Transfer Protocol (HTTP) server breaks the video content into a large number of small chunks and stores them, and the user requests and receives the necessary chunks from the HTTP server at a time as the video plays [49,50]. For smooth video playback, users can do some sequential prefetching. However, this prefetching is intended to reduce the delay in fetching data or to resolve the mismatch between compressed playback and the data fetch speed and is far from reducing the network load. Prefetching is used on a limited basis because fetching future chunks that are not certain to be used can cause unnecessary network load. The method of prefetching and storing chunks slightly behind the content chunk being played by neighboring devices when the wireless network is intermittently under low load during peak hours is referred to in this paper as micro caching. 

**Figure 1:**
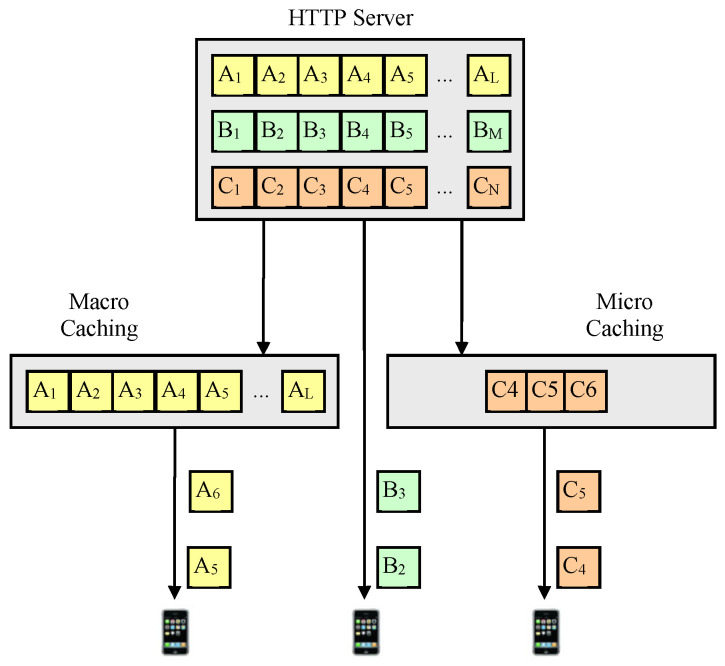
Micro caching.

**Figure 2:**
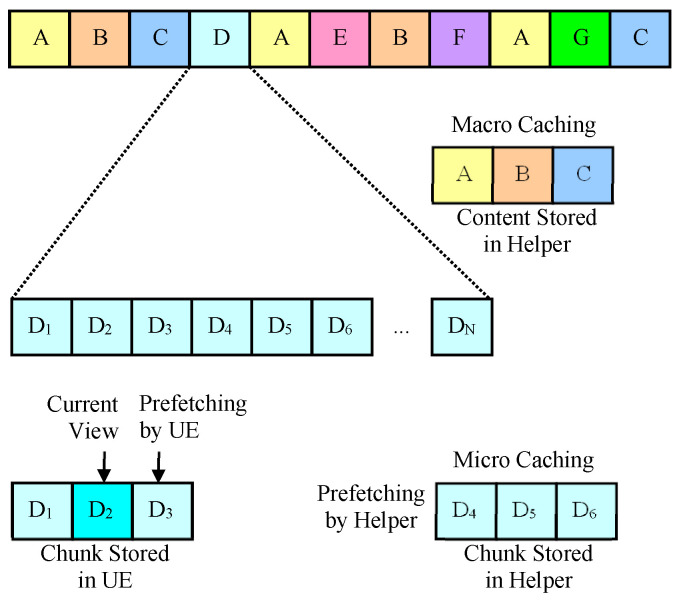
Sequential prefetching.

As shown in Figure 1, in this paper, the method of storing the entire chunks of video content is called macro caching, and the method of storing some video chunks slightly behind the chunk being played by a nearby UE is called micro caching. The fundamental difference between macro caching and micro caching is that macro caching is content-based caching (on the order of minutes or tens of minutes) based on content popularity, while micro caching is chunk-based caching (on the order of seconds) using sequential prefetching, as shown in Figure 2. Assuming that video content consists of a very large number of video chunks and that the video chunks are being watched in sequence, it is possible to predict with relatively high accuracy which chunks will be needed as a user watches a video. Even when one video ends and a new video begins, users tend to watch content that is related to the current content, so it is possible to predict to some extent which chunks will be needed through content recommendation and content prediction algorithms [38,51,52]. The accuracy of sequential prefetching can be determined by how far into the future the prediction is made. Predicting chunks farther in the future can be less accurate because it increases the likelihood that a user will finish watching the current video and start watching another video or skip or stop while watching. In addition, when predicting the distant future, D2D communication may not be possible because the UE leaves the helper’s coverage area.

Micro caching can have very different characteristics from macro caching. In this paper, we investigate the characteristics of micro caching through a rather simple system model to compare it with macro caching. 

A helper can update content when there are intermittent periods of low data demand even during peak hours. If a helper prefetches and stores chunks slightly behind the content chunk being played by the neighboring devices and delivers them when needed, a high hit ratio can be achieved, provided that the users continue to watch the videos and stay within the D2D communication range of the helper. In this paper, it is assumed that the number of helpers is small compared to the number of UEs, so that one helper can deliver content to multiple UEs. It is assumed that the network structure of D2D communication has a star topology and that not too many UEs are connected to a specific helper to avoid congestion in the helper. 

**Figure 3:**
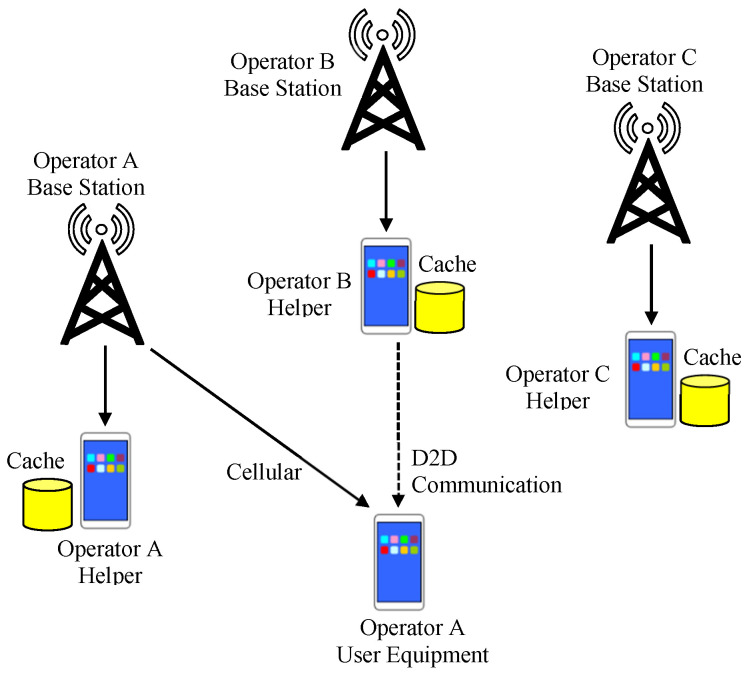
D2D caching for multiple operators.

If the D2D communication can be performed using an unlicensed band, the D2D communication can be performed between devices belonging to different operators [29,30]. Figure 3 illustrates the D2D communication considering multiple operators. If there are multiple operators in a certain region and devices can receive data from helpers of other operators through D2D communication, even if one operator is overloaded, a helper of another operator may be able to perform micro D2D caching for the UEs in the overloaded operator. 

### 2.2. Caching Scenario

Depending on the mobility of devices, they can be categorized as fixed devices that are attached to a specific location, such as Internet of Things (IoT) devices, nomadic devices that move at very low or intermittent speeds, such as pedestrians, and mobile devices that move at high speeds, such as vehicles. When a helper is fixed, only the region in which it is located is considered for caching, while when a helper is mobile at high speed, micro caching can be performed only for UEs that are traveling together with the helper. If there is no UE moving with a mobile helper, micro caching may not be appropriate because UEs will leave the D2D communication range. To simplify the discussion, this paper does not explicitly consider fixed helpers or group mobility and assumes that helpers performing micro caching are nomadic. However, the discussion in this paper can easily be extended to fixed helpers located at a given location or mobile helpers traveling in groups with some UEs. 

Macro caching is generally content-based rather than chunk-based. However, in order to compare the characteristics of micro caching with macro caching, this paper considers macro caching which also stores on a chunk basis. In this case, macro caching and micro caching are both chunk-based, and chunks can be stored based on content popularity in macro caching, while chunk-based sequential prefetching is performed in micro caching. 

**Figure 4:**
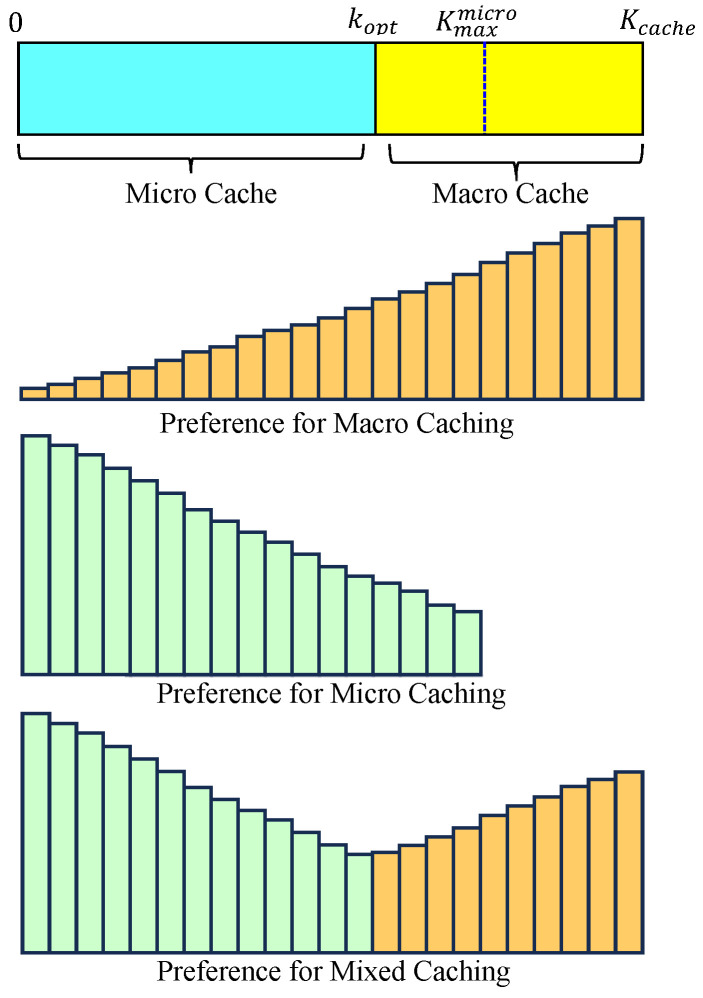
Macro and micro caching areas.

Micro caching is not always better than macro caching, so this paper considers mixed caching, where a cache space is divided into micro and macro cache areas. Consider a helper that divides the cache space into two parts: the macro cache area and the micro cache area, as shown in Figure 4. Before peak hours, the helper considers the popularity of the content and fills the macro cache area in order of decreasing popularity. If the wireless network has an intermittent low load even during peak hours, the macro cache area can be updated by taking into account real-time changes in popularity. For simplicity, this paper does not consider changes in popularity over time, and content is stored in the macro cache area before peak hours and remains in that state during peak hours, so the helper only updates the micro cache area during peak hours when the wireless network is under a low load. 

We assume that helpers use the operator’s spectrum to store content, but the D2D communication between devices uses an unlicensed spectrum, allowing devices belonging to different operators to communicate. The number of helpers is small compared to the number of UEs, and multiple UEs are associated with a UE, forming a star topology. If there are multiple helpers near a UE, the UE is assumed to select and associate with one of the nearby helpers. In particular, when there are helpers from multiple operators in the vicinity, a UE is assumed to associate with the one that is able to perform micro D2D caching by updating the cache. If there is a request for a content chunk from a UE and the helper has the content chunk in the cache, the helper will deliver it to the UE. If the helper does not have the content chunk for a UE, the UE requests the content chunk from the wireless network. If a UE moves out of the helper’s coverage area, or if the wireless network load becomes too congested for the helper to update its cache, the UE can associate with other nearby helpers. 

**Figure 5:**
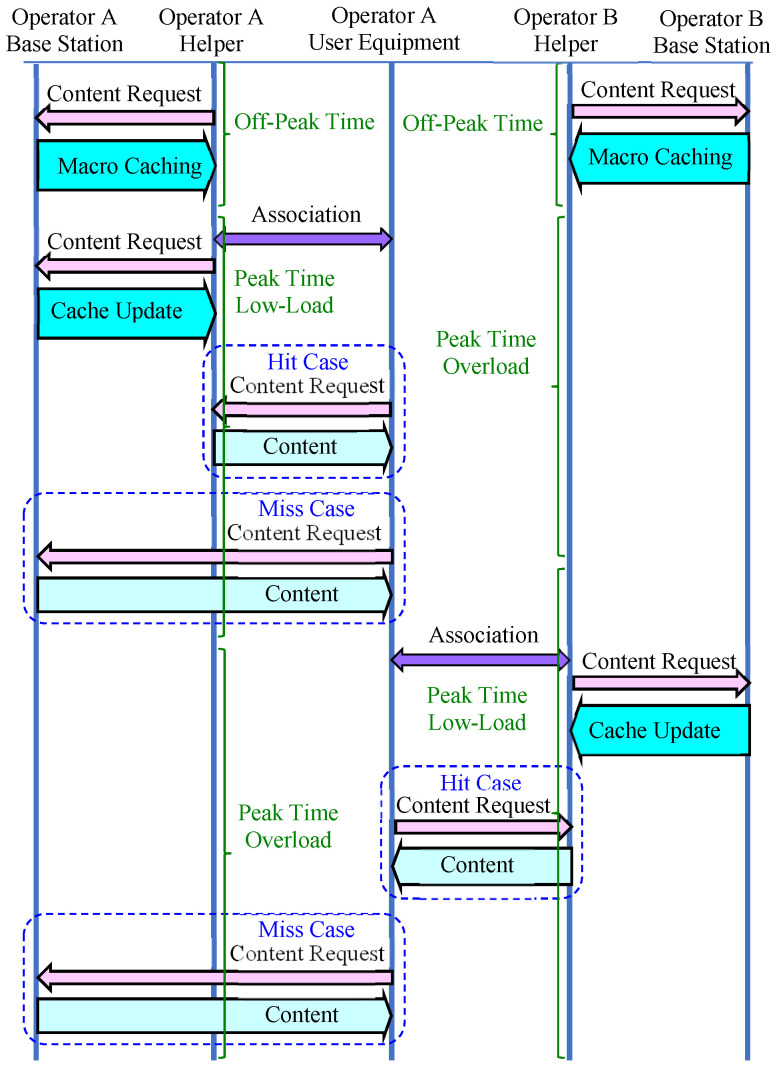
Caching scenario.

Figure 5 illustrates a caching scenario. In a given region, operators and their helpers can be in one of the following states: off-peak time, (peak time) overload, and (peak time) low-load states. In the off-peak time state, a helper stores content chunks in the macro cache area, and in the low-load state, a helper periodically updates content chunks in the micro cache area. In the overload state, no content chunks can be stored or updated. In the peak time states, a UE establishes an association with a nearby helper, and the helper attempts to perform micro D2D caching for the associated UEs. When a UE requests a content chunk, it is delivered via D2D communication if the helper has the chunk in its cache. If the helper does not have the chunk, it is delivered via cellular communication from the base station. When a helper enters the overload state, UEs associated with the helper check for another helper in the low-load state nearby and attempt to establish associations with that helper if necessary. 

Assume that the region under consideration has an area of $A_{total}$ and that there are $N_{operator}$ operators in this region. For simplicity, assume that the helpers of each operator are uniformly distributed and that a UE is associated with one helper at a time. If there are multiple helpers nearby, a UE chooses one of them and establishes an association with that helper. Assume that in the region under consideration, $N_{helper}$ helpers are independently and uniformly distributed for each operator and the D2D radius is $R_{D2D}$. Considering only one operator, the probability that a UE can connect to any helper is

(1) $$P_{helper}^{single}=1-{\left(1-\frac{\pi \;{R_{D2D}}^{2}}{A_{total}}\right)}^{N_{helper}}.$$

The probability that a UE can be associated with one of the helpers of $N_{operator}$ operators is

(2) $$P_{helper}^{multiple}=1-{\left(1-P_{helper}^{single}\right)}^{N_{operator}}.$$

If we assume that the number of helpers is sufficiently large, Equation (1) may become close to 1, and Equation (2) will also converge to 1. In this paper, we assume that these values are close to 1 and do not specifically consider the probability that there is no helper around a UE. When the helper density is very low and there are no helpers around the UE, the effectiveness of D2D caching is greatly reduced, and both macro caching and micro caching become meaningless. In order to provide a simple comparison between micro caching and macro caching when D2D caching is effective, the cases where the UE cannot be associated with any helper are not explicitly considered in this paper. 

Assuming that $N_{UE}$ UEs are independently and uniformly distributed for each operator in the considered area, the average number of UEs connected to a single helper is as follows:

(3) $$N_{device}^{single}=\frac{P_{helper}^{single}N_{UE}}{N_{helper}}\approx \frac{N_{UE}}{N_{helper}}.$$

Assuming that there are $N_{operator}$ operators in the considered region and a UE associates with the helper of the operator with the lowest load, the average number of devices per helper is as follows:

(4) $$N_{device}^{multiple}={N_{operator}N}_{device}^{single}\approx \frac{{N_{operator}N}_{UE}}{N_{helper}}.$$

It is assumed that a UE periodically performs reassociation and, if possible, connects to a helper in the low-load state, and that each helper is tuned to be associated with $N_{device}^{multiple}$ or fewer UEs to avoid the congestion. 

### 2.3. Low-Load State

For the sake of simplicity, let’s assume that video chunks have the same data size and playback time and that the playback time of a chunk is $T_{chunk}$. The state of an operator or a helper can be divided into off-peak time and peak time states. Even during the peak time state, if the helper can perform the content update required for micro D2D caching in a period of less than ${T_{period}T}_{chunk}$, the state is called the (peak time) low-load state. Otherwise, the peak time state is called the (peak time) overload state. Assuming that $N_{device}^{multiple}$ activated UEs are connected to a helper, the UEs request $T_{period}N_{device}^{multiple}$ content chunks between content update periods. 

Each helper can store up to $K_{cache}$ chunks in the cache, and in the low-load state, a helper can update up to $K_{store}$ chunks per each update cycle. In the low-load state, a helper considers the chunks expected to be requested by $N_{device}^{multiple}$ UEs and updates up to the following number of chunks:

(5) $$K_{max}^{micro}=\min\left\{{K_{store},K}_{cache},T_{period}N_{device}^{multiple}\right\}.$$

Each operator may have regions where sufficient data can be supplied by falling within the coverage of high-frequency carriers or by using a large number of antennas at the base station, and there may be regions where sufficient data cannot be supplied. Assuming that, for an operator, the considered region is divided into $N_{region}$ regions according to the probability going into the low-load state, let $P_{i}^{region}\;(i=1,\cdots ,N_{region})$ denote the proportion of each region and $P_{i}^{underload}\;(i=1,\cdots ,N_{region})$ denote the probability of being under a low load in each region. The probability of being under a low load for an opeator is written as

(6) $$P_{underload}^{single}=\sum_{i=1}^{N_{region}}P_{i}^{region}P_{i}^{underload}.$$

The probability of being under a low load can have a large value if a large percentage of the area is covered by high-frequency carriers. Suppose the region under consideration is divided into $N_{region}$ regions independently per operator and each region is under low load independently per operator. The probability that any $N_{operator}$ operators will be under a low load at any given time at any given location is

(7) $$P_{underload}^{multiple}=1-{\left(1-P_{underload}^{single}\right)}^{N_{operator}}.$$

As the number of operators considered increases, the probability that any one of them will be under a low load increases. 

## 3. Hit Ratio of D2D Caching

### 3.1. When to Use Macro Caching Only

Suppose the total number of content chunks is $K_{total}\;\left(\gg K_{cache}\right)$. When a content chunk is requested by a UE, the probability that the chunk is the k-th content chunk $C_{k}^{macro}$ is called $P_{k}^{macro}$, and $C_{k}^{macro}$ is sorted in descending order of $P_{k}^{macro}$. In other words,

(8) $$P_{k}^{macro}\geq P_{k+1}^{macro}\;\;\;\;\left(k=1,\cdots ,K_{cache}-1\right).$$

If the helper’s cache can store $K_{cache}$ chunks and the cache is used only for macro D2D caching, the hit ratio for a request for $N_{device}^{multiple}T_{period}$ content chunks is

(9) $$H_{only}^{macro}=\frac{N_{device}^{multiple}T_{period}\sum_{k=1}^{K_{cache}}P_{k}^{macro}}{N_{device}^{multiple}T_{period}}=\sum_{k=1}^{K_{cache}}P_{k}^{macro}.$$

Because the total number of content chunks is nearly infinite while the cache on a device is not large, macro D2D caching alone may not produce satisfactory results. 

### 3.2. When to Use Micro Caching Only

For $T_{period}N_{device}^{multiple}$ chunks expected to be requested by $N_{device}^{multiple}$ UEs connected to the helper, let $U_{k}$ be the user of the content chunk $C_{k}^{micro}$ and $T_{k}^{use}$ be the time at which it is used. Let $P_{view}\left(u,t\right)(u=1,\cdots ,N_{device}^{multiple},t=1,\cdots ,T_{period})$ be the probability that the UE u continues to view the content at time t. If a UE does not continue to view the content at time t, it is assumed that the UE is viewing other content. $P_{view}(u,t)$ can be related to the characteristics of the user, the characteristics of the content the user is viewing, the viewing time, etc., and can decrease as t increases. Let $P_{coverage}\left(u,t\right)(u=1,\cdots ,N_{device}^{multiple},t=1,\cdots ,T_{period})$ be the probability that UE u remains in the helper’s D2D coverage at time t. Assume that a UE moves out of the helper’s coverage area, another UE moves into the area, and thus there is a constant number of UEs in the coverage area. $P_{coverage}(u,t)$ is related to the relative velocity of the helper and the UE and can become smaller as t increases. The probability that the k-th content chunk $C_{k}^{micro}$ will be used is written as

(10) $$\begin{array}{c} P_{k}^{micro}\equiv P_{coverage}\left(U_{k},T_{k}^{use}\right)P_{view}\left(U_{k},T_{k}^{use}\right)\\ \left(k=1,\cdots ,T_{period}N_{device}^{multiple}-1\right). \end{array}$$

Suppose $C_{k}^{micro}$ is sorted in descending order of $P_{k}^{micro}$, in other words,

(11) $$P_{k}^{micro}\geq P_{k+1}^{micro}\;\;\;\;\left(k=1,\cdots ,T_{period}N_{device}^{multiple}-1\right).$$

Consider the case where $K_{store}$ and $T_{period}N_{device}^{multiple}$ are greater than $K_{cache}$, i.e., $K_{max}^{micro}=K_{cache}$ in Equation (5). If the cache is used only as micro D2D caching, the hit ratio is written as

(12) $$H_{only}^{micro}=\frac{P_{underload}^{multiple}}{N_{device}^{multiple}T_{period}}\sum_{k=1}^{K_{cache}}P_{k}^{micro}.$$

### 3.3. When to Maximize Micro Caching

This time, consider the case where $K_{store}$ or $T_{period}N_{device}^{multiple}$ is smaller than $K_{cache}$, i.e., $K_{max}^{micro}<K_{cache}$. Even if the cache is used as much as possible as micro D2D caching, if the case size $K_{cache}$ is larger than the maximum considered micro cache size $K_{max}^{micro}$, the remaining $K_{cache}-K_{max}^{micro}$ area can be used for macro D2D caching. When $K_{max}^{micro}$ area is used for micro D2D caching, the hit ratio for that portion is

(13) $$H_{max}^{micro}=\frac{P_{underload}^{multiple}}{N_{device}^{multiple}T_{period}}\sum_{k=1}^{K_{max}^{micro}}P_{k}^{micro}.$$

The remaining $K_{cache}-K_{max}^{micro}$ area can be used for macro D2D caching, and the hit ratio of the macro D2D caching portion is

(14) $$H_{min}^{macro}=\sum_{k=1}^{K_{cache}-K_{max}^{micro}}P_{k}^{macro}.$$

The hit ratio of micro D2D caching can be calculated by considering both the micro caching part and the macro caching part. If micro caching and macro caching are independent, the cache hit ratio is written as follows:

(15) $$\begin{array}{c} H_{max}^{mixed}=\frac{\sum_{k=1}^{K_{max}^{micro}}\left(P_{underload}^{multiple}P_{k}^{micro}+\left(1-P_{underload}^{multiple}P_{k}^{micro}\right)H_{min}^{macro}\right)}{T_{period}N_{device}^{multiple}}\\ +\left(1-\frac{K_{max}^{micro}}{T_{period}N_{device}^{multiple}}\right)H_{min}^{macro}\\ =\frac{\sum_{k=1}^{K_{max}^{micro}}\left(P_{underload}^{multiple}P_{k}^{micro}\left(1-H_{min}^{macro}\right)\right)}{T_{period}N_{device}^{multiple}}+H_{min}^{macro}\\ =H_{max}^{micro}+H_{min}^{macro}-H_{max}^{micro}H_{min}^{macro}. \end{array}$$

If $K_{store}$ and $T_{period}N_{device}^{multiple}$ are greater than $K_{cache}$, then $K_{max}^{micro}$ becomes $K_{cache}$, so $H_{max}^{micro}$ in Equation (15) becomes $H_{only}^{micro}$ and $H_{min}^{macro}$ becomes zero. Therefore, Equation (15) can be considered as a general case including Equation (12).

### 3.4. When to Use the Right Ratio

In the previous subsection, the helper cache space was used as much as possible as a micro cache area, and the remaining area was used as a macro cache area. However, micro caching is not always superior to macro caching, so it may be necessary to split the two areas appropriately. Figure 4 shows that the cache area is divided into a micro cache area and a macro cache area, where the micro cache area stores content chunks with high preference for micro caching and the macro cache area stores content chunks with high preference for macro caching. It may be more efficient to divide the cache area into appropriate proportions than to store only the highly favored content chunks for macro caching or, conversely, only the highly favored content chunks for micro caching. 

Suppose a helper’s cache space is divided into micro and macro cache areas. Assuming that the helper is nomadic, it does not know in advance which region it will be in during peak hours, so the ratio of micro to macro cache areas must be determined in advance, regardless of the helper’s current location. Based on the pre-determined ratio, popular content is stored in the macro cache before peak hours, and chunks are updated for micro D2D caching when the helper’s operator is under low load. 

Let $k_{0}\;\left(0\leq k_{0}\leq K_{max}^{micro}\right)$ be the number of chunks for micro caching and $K_{cache}-k_{0}$ be the number of chunks for macro caching among the $K_{cache}$ content chunks stored in the helper’s cache. The helper caches content chunks $C_{k}^{macro}$ from 1 to $K_{cache}-k_{0}$ before peak hours. In this case, the hit ratio of the macro cache area alone is as follows:

(16) $$H^{macro}\left(k_{0}\right)=\sum_{k=1}^{K_{cache}-k_{0}}P_{k}^{macro}\;\;\;\left(0\leq k_{0}\leq K_{max}^{micro}\right).$$

The helper periodically updates the micro cache area when the operator is under low load during peak hours. The hit ratio of the micro cache area alone is as follows:

(17) $$H^{micro}\left(k_{0}\right)=\frac{P_{underload}^{multiple}}{N_{device}^{multiple}T_{period}}\sum_{k=1}^{k_{0}}P_{k}^{micro}\;\;\;\;\;\left(0\leq k_{0}\leq K_{max}^{micro}\right).$$

When micro caching and macro caching are independent, the cache hit ratio is written as:

(18) $$\begin{array}{c} H^{mixed}\left(k_{0}\right)=H_{\;}^{micro}\left(k_{0}\right)+H_{\;}^{macro}\left(k_{0}\right)-H_{\;}^{micro}\left(k_{0}\right)H_{\;}^{macro}\left(k_{0}\right)\;\;\;\\ \left(0\leq k_{0}\leq K_{max}^{micro}\right). \end{array}$$

The optimal value of $k_{0}$ is determined such that Equation (18) is maximized, i.e.,

(19) $$H_{opt}^{mixed}\equiv \underset{k_{0}}{\max}H^{mixed}\left(k_{0}\right),$$

(20) $$k_{opt}\equiv \underset{k_{0}}{\mathrm{argmax}}H^{mixed}\left(k_{0}\right).$$

By calculating all $k_{0}$ values from zero to $K_{max}^{micro}$, the optimal value can be found. However, let’s take a quick look at the properties of $k_{opt}$. If $H_{\;}^{micro}\left(k_{opt}\right)$ and $H_{\;}^{macro}\left(k_{opt}\right)$ are sufficiently small compared to 1, then $H_{\;}^{micro}\left(k_{opt}\right)H_{\;}^{macro}\left(k_{opt}\right)$ can be negligible in Equation (18). Consider the approximate hit ratio:

(21) $$\begin{array}{c} {\tilde{H}}^{mixed}\left(k_{0}\right)\equiv H_{\;}^{micro}\left(k_{0}\right)+H_{\;}^{macro}\left(k_{0}\right)\\ =\frac{P_{underload}^{multiple}}{N_{device}^{multiple}T_{period}}\sum_{k=1}^{k_{0}}P_{k}^{micro}+\sum_{k=1}^{K_{cache}-k_{0}}P_{k}^{macro}. \end{array}$$

Suppose $K_{1}$ satisfies the following equation:

(22) $$\begin{array}{c} P_{K_{cache}-K_{1}+1}^{macro}\leq \frac{P_{underload}^{multiple}}{N_{device}^{multiple}T_{period}}P_{K_{1}}^{micro},\\ \frac{P_{underload}^{multiple}}{N_{device}^{multiple}T_{period}}P_{K_{1}+1}^{micro}\leq P_{K_{cache}-K_{1}}^{micro}. \end{array}$$

When $K_{1}\leq K_{max}^{micro}$, the following is satisfied for $k_{0}(<K_{1})$:

(23) $$\begin{array}{c} {\tilde{H}}^{mixed}\left(K_{1}\right)-{\tilde{H}}^{mixed}\left(k_{0}\right)=\frac{P_{underload}^{multiple}}{N_{device}^{multiple}T_{period}}\sum_{k=k_{0}+1}^{K_{1}}P_{k}^{micro}-\sum_{k=K_{cache}-K_{1}+1}^{K_{cache}-k_{0}}P_{k}^{macro}\\ \geq \frac{P_{underload}^{multiple}}{N_{device}^{multiple}T_{period}}\left(\;K_{1}-k_{0}\right)P_{K_{1}}^{micro}-\left(\;K_{1}-k_{0}\right)P_{K_{cache}-K_{1}+1}^{macro}\\ =\left(\;K_{1}-k_{0}\right)\left(\frac{P_{underload}^{multiple}}{N_{device}^{multiple}T_{period}}P_{K_{1}}^{micro}-P_{K_{cache}-K_{1}+1}^{macro}\right)\geq 0. \end{array}$$

When $K_{1}\leq K_{max}^{micro}$, the following is satisfied for $k_{0}(>K_{1})$:

(24) $$\begin{array}{c} {\tilde{H}}^{mixed}\left(K_{1}\right)-{\tilde{H}}^{mixed}\left(k_{0}\right)=\sum_{k=K_{cache}-k_{0}+1}^{K_{cache}-K_{1}}P_{k}^{macro}-\frac{P_{underload}^{multiple}}{N_{device}^{multiple}T_{period}}\sum_{k=K_{1}+1}^{k_{0}}P_{k}^{micro}\;\\ \geq \left(\;k_{0}-K_{1}\right)P_{K_{cache}-K_{1}}^{macro}-\frac{P_{underload}^{multiple}}{N_{device}^{multiple}T_{period}}\left(\;k_{0}-K_{1}\right)P_{K_{1}+1}^{micro}\\ =\left(\;k_{0}-K_{1}\right)\left(P_{K_{cache}-K_{1}}^{macro}-\frac{P_{underload}^{multiple}}{N_{device}^{multiple}T_{period}}P_{K_{1}+1}^{micro}\right)\geq 0.\; \end{array}$$

When $K_{1}>K_{max}^{micro}$, the following is satisfied for $k_{0}(<K_{max}^{micro})$:

(25) $$\begin{array}{c} {\tilde{H}}^{mixed}\left(K_{max}^{micro}\right)-{\tilde{H}}^{mixed}\left(k_{0}\right)=\frac{P_{underload}^{multiple}}{N_{device}^{multiple}T_{period}}\sum_{k=k_{0}+1}^{K_{max}^{micro}}P_{k}^{micro}-\sum_{k=K_{cache}-K_{max}^{micro}+1}^{K_{cache}-k_{0}}P_{k}^{macro}\\ \geq \frac{P_{underload}^{multiple}}{N_{device}^{multiple}T_{period}}\left(\;K_{max}^{micro}-k_{0}\right)P_{K_{max}^{micro}}^{micro}-\left(\;K_{max}^{micro}-k_{0}\right)P_{K_{cache}-K_{max}^{micro}+1}^{macro}\\ =\left(\;K_{max}^{micro}-k_{0}\right)\left(\frac{P_{underload}^{multiple}}{N_{device}^{multiple}T_{period}}P_{K_{max}^{micro}}^{micro}-P_{K_{cache}-K_{max}^{micro}+1}^{macro}\right)\geq 0.\; \end{array}$$

Therefore, the value of $k_{0}$ that maximizes ${\tilde{H}}^{mixed}\left(k_{0}\right)$ is

(26) $${\tilde{k}}_{opt}\equiv \underset{k_{0}}{\mathrm{argmax}}{\tilde{H}}^{mixed}\left(k_{0}\right)=\min\left\{K_{max}^{micro},K_{1}\right\}.$$

In Equations (10), (22) and (26), ${\tilde{k}}_{opt}$ may increase as the probability of being under low load increases, the mobility of devices decreases, and the probability of continuing to view content increases. As the number of UEs decreases or the update cycle decreases, ${\tilde{k}}_{opt}$ can increase if $K_{max}^{micro}$ is much larger than $K_{1}$. If $K_{max}^{micro}$ is not sufficiently larger than $K_{1}$, then reducing the number of UEs or the update cycle may reduce $K_{max}^{micro}$, resulting in a smaller ${\tilde{k}}_{opt}$. 

The performance gain of $H_{opt}^{mixed}$ over $H_{only}^{macro}$, which is measured as the difference between $H_{opt}^{mixed}$ and $H_{only}^{macro}$, is approximated as follows:

(27) $$\begin{array}{c} H_{opt}^{mixed}-H_{only}^{macro}\approx {\tilde{H}}^{mixed}\left({\tilde{k}}_{opt}\right)-H_{only}^{macro}\\ =H_{\;}^{micro}\left({\tilde{k}}_{opt}\right)-\left(H_{only}^{macro}-H_{\;}^{macro}\left({\tilde{k}}_{opt}\right)\right)\\ =\frac{P_{underload}^{multiple}}{N_{device}^{multiple}T_{period}}\sum_{k=1}^{\min\left\{K_{max}^{micro},K_{1}\right\}}P_{k}^{micro}-\sum_{k=K_{cache}-\min\left\{K_{max}^{micro},K_{1}\right\}+1}^{K_{cache}}P_{k}^{macro}. \end{array}$$

The performance gain is determined by how much larger the hit ratio of the micro cache area is compared to the hit ratio when that area is used for macro caching. The greater the probability of being under low load, the less mobile the devices, the more likely the video is to be viewed continuously, and the greater the performance gain. 

## 4. Numerical Results

In this section, we examine the hit ratio and the ratio of the micro cache area to the cache size when using mixed caching. In the simulation, the total number of content chunks, $K_{total}$, is 1,000,000, the macro preferences of the content chunks have a Zipf distribution with Zipf coefficient λ = 0.8, and the number of operators, $N_{operator}$, is 4. Each operator has two types of regions, one for a high-frequency carrier coverage and the other for a low-frequency carrier coverage, and the proportions and low-load state probabilities of the regions are $P_{1}^{region}$ = 0.4, $P_{2}^{region}$ = 0.6, $P_{1}^{underload}$ = 0.7, and $P_{2}^{underload}$ = 0.1. Region 1 may refer to a high-frequency carrier region with a high probability of being under a low load and Region 2 may refer to the low-frequency carrier region with a low probability of being under a low load. 

The number of devices of each operator in the D2D area, $N_{device}^{single}$, is five, and therefore the number of UEs supported by a helper, $N_{device}^{multiple}$, is limited to 20. The cache update cycle, $T_{period}$, is 20, the cache size of a helper, $K_{cache}$, is 400, and the number of chunks that can be updated per update cycle, $K_{store}$, is 400. Since $N_{device}^{multiple}T_{period}$, $K_{cache}$, and $K_{store}$ are all 400, $K_{max}^{micro}$ is also 400. The probability that UE u is still viewing content at time t is:

(28) $$\begin{array}{c} P_{\;view}\left(u,t\right)=\alpha_{view}\exp\left(-\beta_{view}t\right)\;\;\\ \left(u=1,\cdots ,N_{device}^{multiple},\;t=1,\cdots ,T_{period}\right)\; \end{array}$$

where $\alpha_{view}$ is 0.8 and $\beta_{view}$ is between 0 and 0.2. Assuming that a UE is mobile with a probability of $P_{mobile}$, the probability that a UE will remain within the helper’s coverage, $P_{\;coverage}(u,t)$, is

(29) $$\begin{array}{c} P_{\;coverage}\left(u,t\right)=\left\{\begin{array}{c} 1\;\;\;\;\;\;\;\;\;\;\;\;\;\;\;\;\;\;if\;u\leq N_{device}^{multiple}\left(1-P_{mobile}\right)\\ \alpha_{coverage}\;\;\;\;\;\;\;\exp\left(-\beta_{coverage}t\right)\;\;\;\;otherwise \end{array}\right.\\ \left(u=1,\cdots ,N_{device}^{multiple},t=1,\cdots ,T_{period}\right). \end{array}$$

where $P_{mobile}$ is 0.25, $\alpha_{coverage}$ is 0.8, and $\beta_{coverage}$ is 0.2. Each experiment shows two figures: the first figure shows the hit ratios $H_{only}^{macro}$ of macro caching and $H_{opt}^{mixed}$ of mixed caching, and the second figure shows the proportion of the micro cache area in the cache $k_{opt}/K_{cache}$ and the approximate ratio ${\tilde{k}}_{opt}/K_{cache}$. In most cases in the experiments, $K_{max}^{micro}=K_{cache}$ and the maximum value of the micro caching ratio is one. The optimal results were found through an exhaustive search. The simulation parameters are summarized in Table 1. 

**Figure 6:**
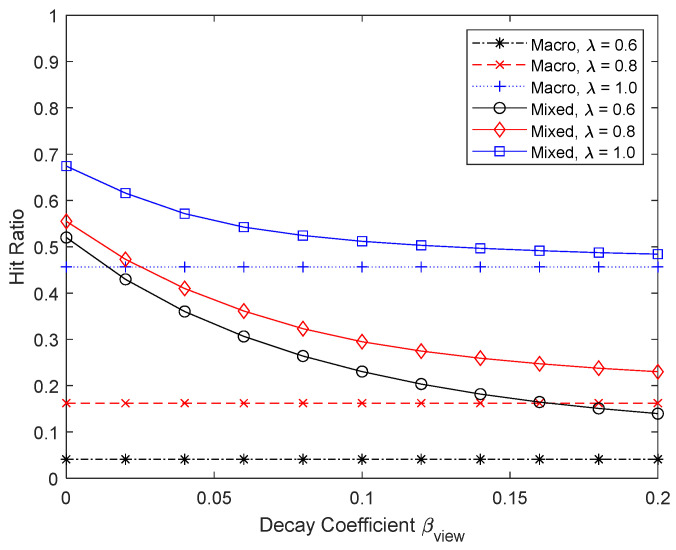
Hit ratio depending on the Zipf coefficient.

**Figure 7:**
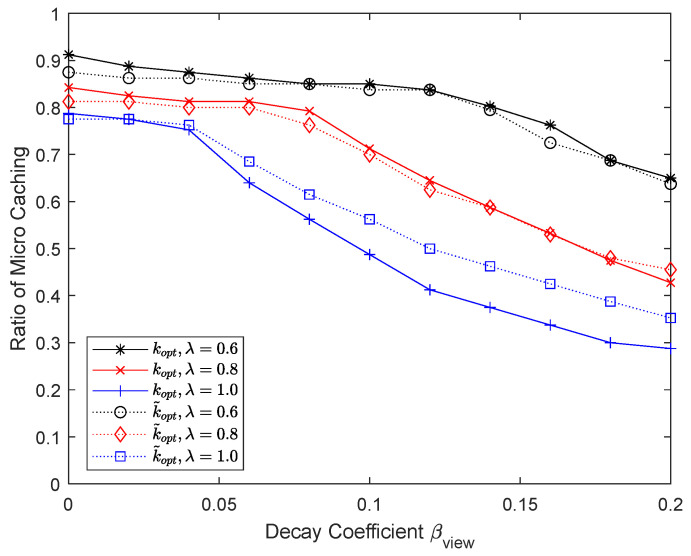
Proportion of micro cache area depending on the Zipf coefficient.

Figure 6 and Figure 7 show the numerical results when the Zipf coefficient is 0.6, 0.8, and 1.0. The maximum number of chunks that can be micro D2D cached, $K_{max}^{micro}$, is 400, so the maximum micro D2D caching ratio is one. When the Zipf coefficient is large, a high hit ratio can be achieved even with a small cache size, but as the Zipf coefficient becomes smaller, the performance of macro caching deteriorates. By using a portion of the cache as a micro cache area, a significant performance improvement can be achieved, especially when the Zipf coefficient is not large and thus the performance of macro caching alone is not satisfactory with a limited cache size. As $\beta_{view}$ increases, the probability that content chunks stored in the micro cache will not be used increases, so the effectiveness of micro caching decreases and the proportion of the micro cache area also decreases. When the Zipf coefficient is very large and the cache hit ratio is high, $H_{\;}^{micro}\left(k_{opt}\right)H_{macro}\left(k_{opt}\right)$ cannot be ignored and ${\tilde{k}}_{opt}$ is somewhat different from $k_{opt}$. In other cases, however, ${\tilde{k}}_{opt}$ has a similar value to $k_{opt}$.

**Figure 8:**
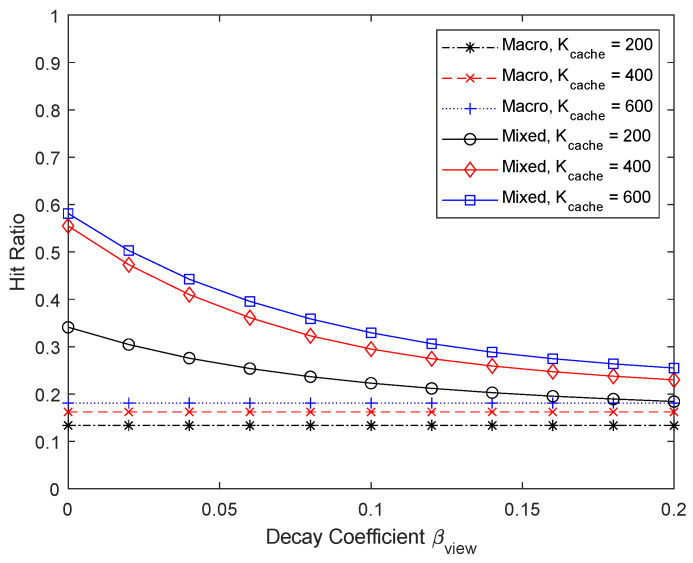
Hit ratio depending on the case size.

**Figure 9:**
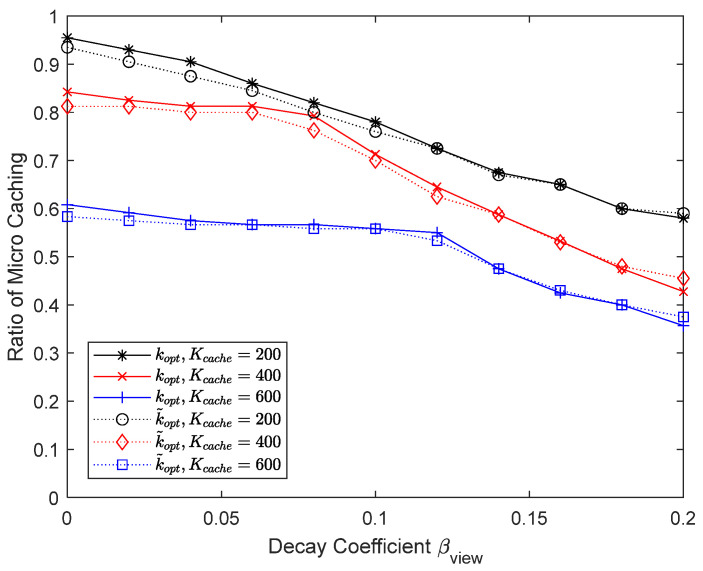
Proportion of micro cache area depending on the cache size.

Figure 8 and Figure 9 show the experimental results when the cache size is varied to 200, 400, and 600. The number of chunks considered for micro caching is 400, so even if the cache size is increased to 600, the micro cache area cannot be increased beyond 400 and only the macro cache area becomes larger. In this case, the proportion of the micro cache area is less than 2/3. Figure 8 shows that the performance improvement from increasing the macro cache area from 400 to 600 is not significant. On the other hand, when the cache size is reduced from 400 to 200, the performance drops significantly because there is not enough space for micro caching. When only macro caching is used, it can be seen that the performance difference depending on the cache size is relatively small. When using micro caching, it is important to ensure that enough cache space is available for micro caching and the performance improvement is not significant even if the cache size becomes much larger than the maximum micro cache size. In this simulation, there are no cases with very large Zipf coefficients, so ${\tilde{k}}_{opt}$ has a similar value to $k_{opt}$.

**Figure 10:**
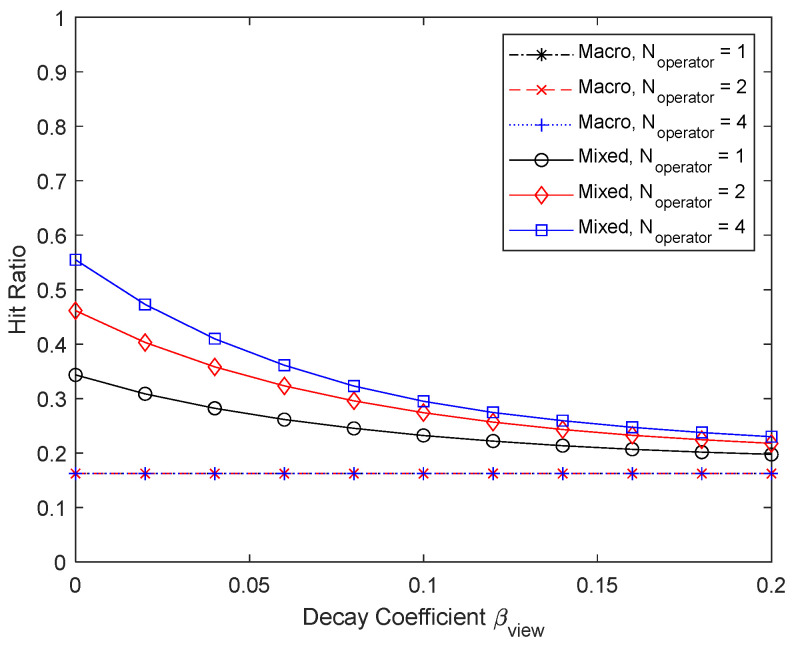
Hit ratio depending on the number of operators.

**Figure 11:**
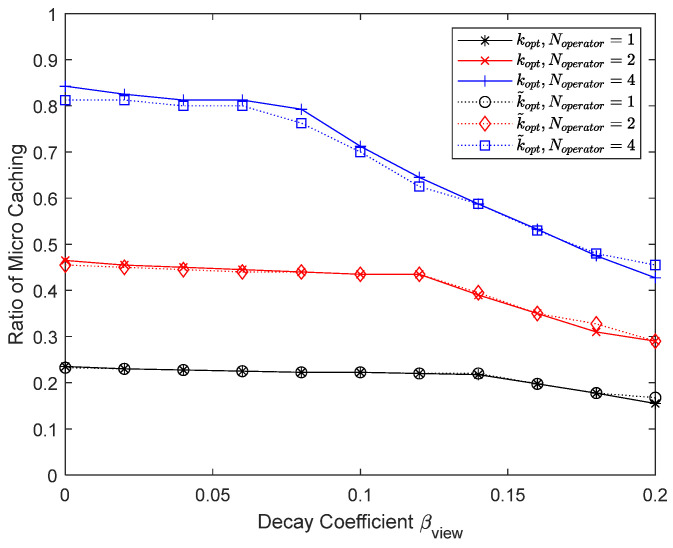
Proportion of micro cache area depending on the number of operators.

Figure 10 and Figure 11 show the results when the number of operators is varied to 1, 2, and 4. Since it is assumed that a UE is associated with only one helper at a time, the helpers do not cooperate to store different content chunks from each other, and there is no performance improvement for macro caching as the number of operators increases. In this paper, we do not consider that helpers cooperate with each other, but as the number of operators increases, the probability that at least one of the operators will be under a low load increases, thereby improving the performance of micro caching. When the number of operators is 1 and 2, the number of chunks for micro caching is 100 and 200, respectively, so the proportion of the micro cache area is less than 1/4 and 1/2, respectively. In these cases, the remaining area in the cache is used for macro caching to benefit from improved caching performance, but the performance of mixed caching deteriorates due to the low probability that at least one of the operators will be under a low load. 

**Figure 12:**
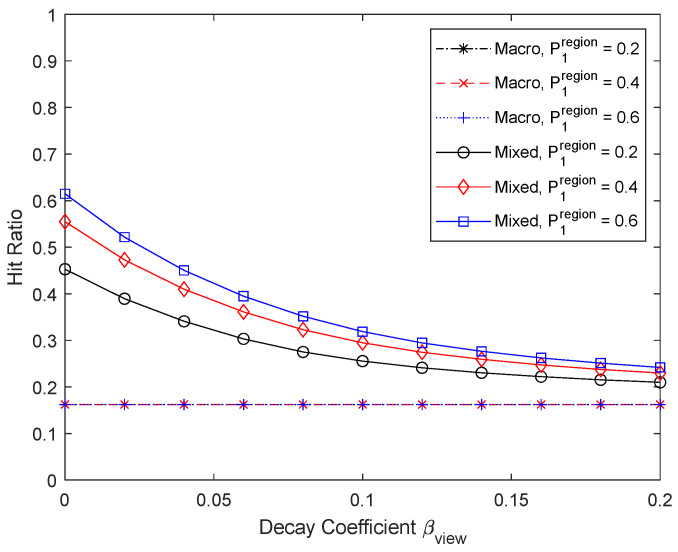
Hit ratio depending on the percentage of the high-frequency carrier region.

**Figure 13:**
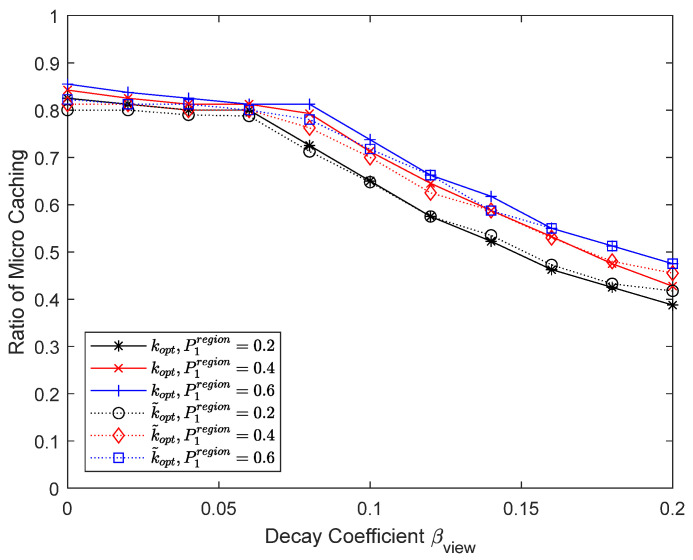
Proportion of micro cache area depending on the percentage of the high-frequency carrier region.

Figure 12 and Figure 13 show the results when the percentage of the high-frequency carrier region is varied to 0.2, 0.4, and 0.6. As the percentage of the high-frequency carrier region increases, cache updates become more frequent, the effectiveness of micro caching increases, and the proportion of the micro cache area also increases. For macro caching, we do not consider temporal changes in content preferences, so there is no performance improvement by cache updates, and therefore there is no performance difference depending on the proportion of the high-frequency carrier region. If we consider the changes in content preferences over time in macro caching, increasing the high-frequency carrier region will allow cache updates during peak hours, resulting in an improvement in macro caching performance.

**Figure 14:**
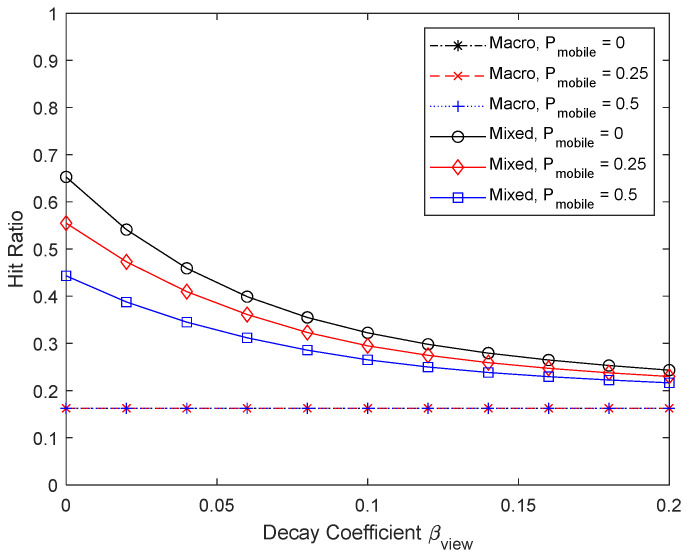
Hit ratio depending on the percentage of mobile devices.

**Figure 15:**
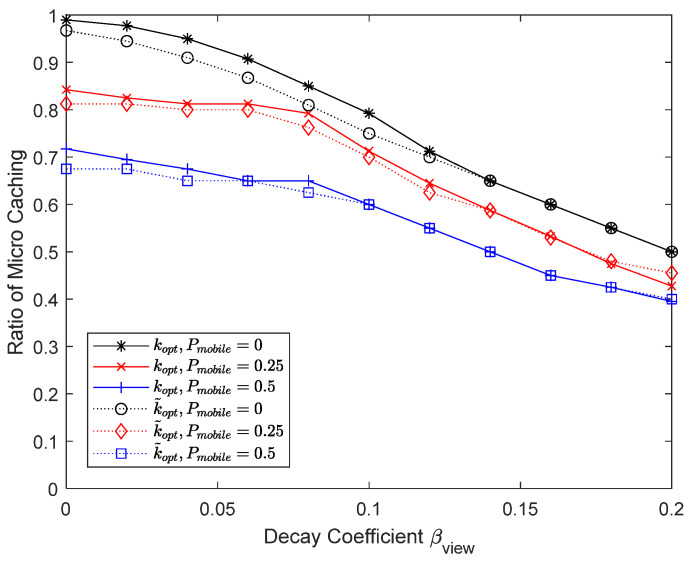
Proportion of micro cache area depending on the percentage of mobile devices.

Figure 14 and Figure 15 show the performance by changing the proportion of mobile UEs to 0, 0.25, and 0.5. Micro caching becomes less effective as more UEs become mobile, making it less likely that the UEs will stay within the helper’s coverage area. Since it is not effective to perform micro caching for mobile devices, the percentage of micro cache area in the cache decreases with a large proportion of mobile devices. To enable micro caching for fast moving UEs, it may be necessary to have a mobile helper that moves together with the moving UEs.

**Figure 16:**
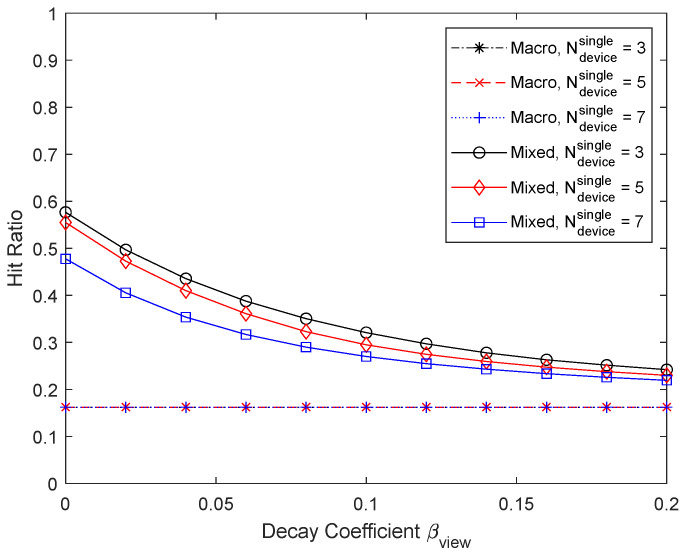
Hit ratio depending on the number of devices per operator.

**Figure 17:**
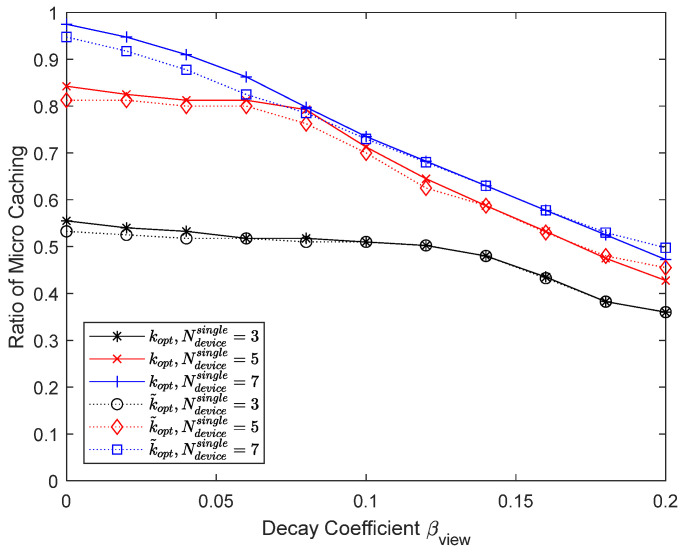
Proportion of micro cache area depending on the number of devices per operator.

Figure 16 and Figure 17 show the results when the number of UEs in D2D coverage in each operator is varied to 3, 5, and 7. Since the number of operators is 4, the number of UEs that can be associated with a helper is 12, 20, and 28, respectively, and the number of chunks updated for micro caching is 240, 400, and 560, respectively. The cache size is 400, so the proportion of the micro cache area is less than 0.6 when the number of UEs per operator is 3. In this case, the micro cache area is small, and the remaining part can be used for macro caching, resulting in a slightly larger hit ratio, but the difference is not significant. On the other hand, if the number of UEs per operator is 7, the hit ratio drops significantly because the micro cache area is insufficient. For micro caching to work well, there must be enough cache area to store the chunks that the UEs are expected to request.

**Figure 18:**
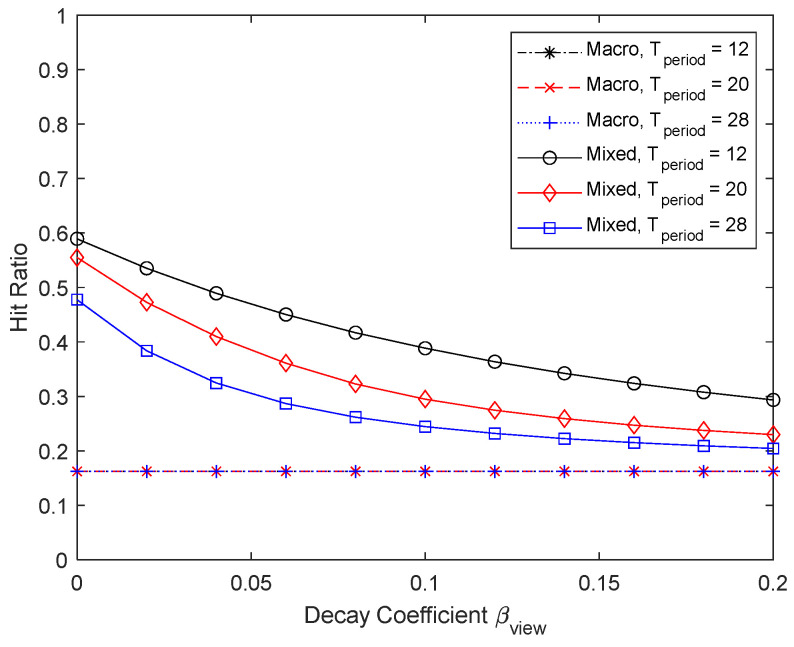
Hit ratio depending on the cache update cycle.

**Figure 19:**
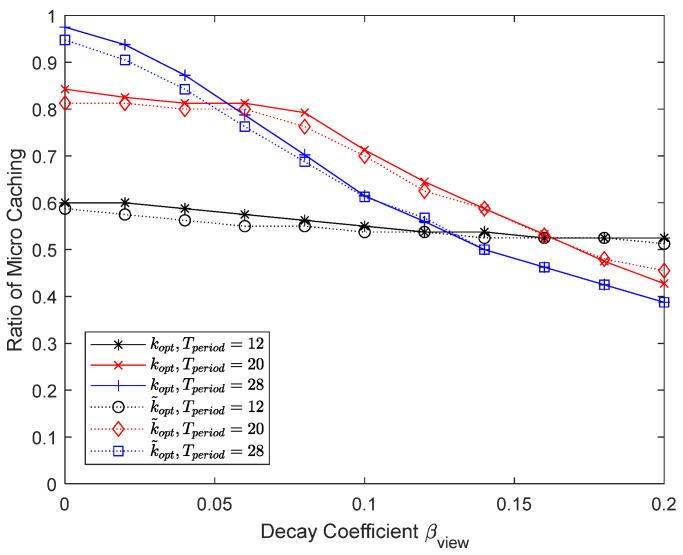
Proportion of micro cache area depending on the cache update cycle.

Figure 18 and Figure 19 show the results when the content update cycle is changed to 12, 20, and 28. As in the case of varying the number of UEs in Figure 16 and Figure 17, the number of chunks by varying the content update cycle is 240, 400, and 560, respectively. However, the hit ratios are somewhat different from the results in Figure 16, especially when $\beta_{view}$ increases. When the cache update cycle is shortened, the hit ratio is less affected, even with a large $\beta_{view}$. Conversely, as the cache update cycle increases, micro caching becomes less effective, and the percentage of the micro cache area decreases as $\beta_{view}$ increases. It can be seen that micro caching benefits from a shorter cache update cycle.

**Figure 20:**
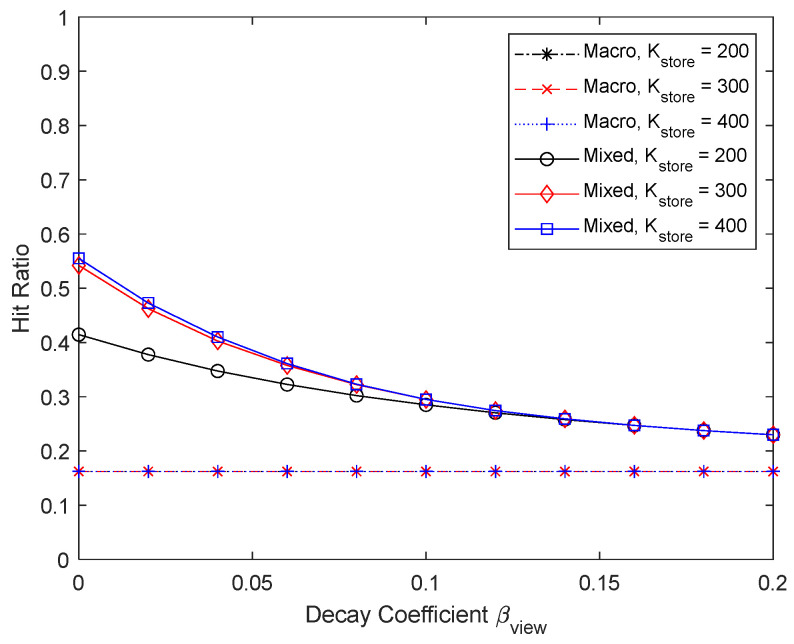
Hit ratio depending on the number of chunks that can be updated.

**Figure 21:**
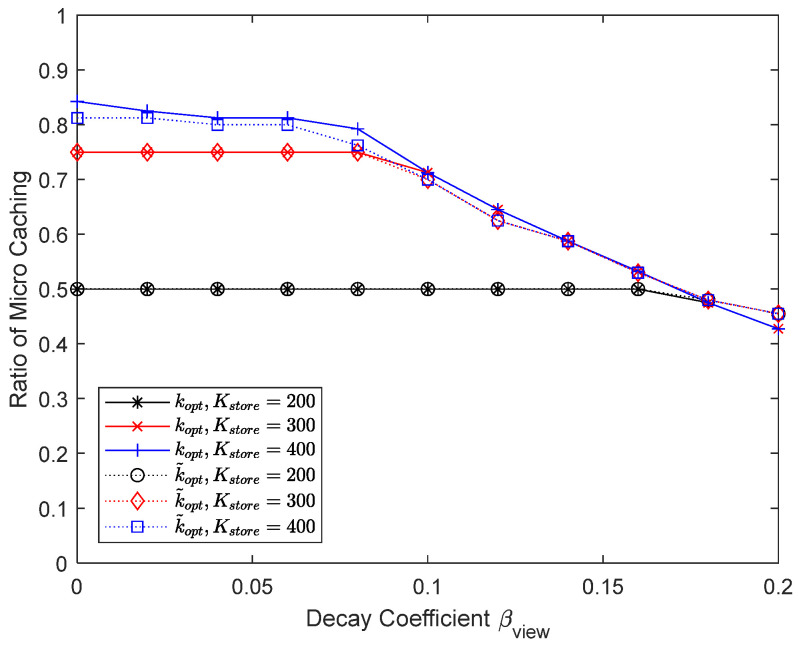
Proportion of micro cache area depending on the number of chunks that can be updated.

Figure 20 and Figure 21 show the results of varying the maximum number of chunks that can be updated per content update cycle to 200, 300, and 400. The maximum percentage of micro caching is 0.5 when the number of chunks that can be updated is 200 and 0.75 when it is 300. Since micro caching is less effective for mobile UEs anyway, there is no need to store all the content chunks that are expected to be requested by mobile UEs, and reducing the number of chunks that can be updated to 300 does not have a significant impact on the performance. However, if the number of chunks that can be updated becomes very small, the hit ratio will not be satisfactory. Being able to update a sufficient number of chunks when updating the cache is critical for micro caching to work well. 

## 5. Conclusions

In this paper, we investigated the performance and effectiveness of micro D2D caching when there are multiple operators, devices can communicate with devices of other operators, and operators are under a low load independently of each other. Assuming that the cache can be updated intermittently even during peak hours and that the time for the operator to become under a low load is independent, a significant performance improvement can be achieved by micro D2D caching. In this paper, it is shown that using a mixture of micro and macro caching, by dividing helpers’ cache space into micro and macro cache areas, can result in a significant performance improvement over macro caching alone. In particular, the use of micro D2D caching can provide maximum benefit in the following cases:

- When macro caching alone does not provide sufficient performance.
- When there is sufficient storage space in a helper for chunk prefetching.
- When there are multiple operators and the operators are under a low load independently of each other.
- If there are enough high-frequency carrier regions that a helper’s cache can be updated intermittently even during peak hours.
- If the proportion of mobile devices is small.
- If users are likely to continue viewing the content they are currently viewing.
- If the content update cycle is short.
- If a sufficient number of chunks can be updated per content update cycle.

The mixed D2D caching method proposed in this paper, which is a combination of micro and macro caching, can be used in conjunction with conventional methods to improve the performance of macro D2D caching and can be further improved by using a combination of different techniques. For example, when predicting the mobility pattern of devices or considering recommendation systems, it is possible to benefit from both micro and macro caching, and further research is needed concerning how to maximize the synergies between these techniques. 

For simplicity, this paper assumes that a UE is associated with a single helper at any given time. However, further performance improvements can be achieved if a UE can be associated with multiple nearby helpers, which can increase the effective storage space of the cooperative helpers. In the future, research is needed on how to store and update content chunks when multiple helpers cooperate to perform both micro caching and macro caching.

## Figures and Tables

**Table 1:** Simulation parameters.

Parameter	Value
The total number of content chunks ($K_{total}$)	1,000,000
Zipf coefficient for macro caching (λ)	0.8
The number of operators $(N_{operator}$)	4
The proportion of region 1 ($P_{1}^{region}$)	0.4
The proportion of region 2 ($P_{2}^{region}$)	0.6
The low-load state probability of region 1 ($P_{1}^{underload}$)	0.7
The low-load state probability of region 2 ($P_{2}^{underload}$)	0.1
The number of devices per each operator in the D2D area ($N_{device}^{single}$)	5
The number of UEs supported by a helper ($N_{device}^{multiple}$)	20
The cache update cycle ($T_{period}$)	20
The cache size of a helper ($K_{cache}$)	400
The number of chunks that can be updated per content update cycle ($K_{store}$)	400
The probabilty that a UE is mobile ($P_{mobile}$)	0.25

## Data Availability

Data are contained within the article.
